# Large Pyoderma Gangrenosum-Like Ulcers: A Rare Presentation of Granulomatosis with Polyangiitis

**DOI:** 10.1155/2014/850364

**Published:** 2014-05-18

**Authors:** Basheer Tashtoush, Roya Memarpour, Yasmin Johnston, Jose Ramirez

**Affiliations:** ^1^Department of Pulmonary and Critical Care Medicine, Cleveland Clinic Florida, 2950 Cleveland Clinic Boulevard, Weston, FL 33331, USA; ^2^Department of Clinical Pathology, Cleveland Clinic Florida, 2950 Cleveland Clinic Boulevard, Weston, FL 33331, USA

## Abstract

Granulomatosis with polyangiitis (GPA), formerly known as Wegener's granulomatosis (WG), is a rare systemic vasculitis that classically manifests as necrotizing granulomas of the upper and lower respiratory tract, kidneys, and blood vessels; however, it may affect any organ system, including the skin. Cutaneous manifestations occur in up to 45% of patients during the disease course, and are the presenting feature in 9% to 14% of patients. The most common skin lesion specific to GPA is palpable purpura, with the histopathologic correlate of leukocytoclastic vasculitis. However, a wide range of clinical and histologic features may be seen. We herein report a case of a previously healthy 52-year-old Caucasian man who presented with multiple progressively enlarging painful ulcers on his face, upper extremities, back, and abdomen over a two-month period. Skin biopsies revealed pyoderma gangrenosum-like features. Serological tests were positive for PR3/c-ANCA. Six months later, the patient developed recurrent episodes of sinusitis associated with nasal bleeds and eventually nasal septum perforation. Despite aggressive treatment with Cyclophosphamide and steroids over one year, the patient had persistent nonhealing large ulcers and developed multiple lung nodules with cavitary lesions.

## 1. Introduction

The hallmarks of GPA are systemic necrotizing vasculitis, necrotising granulomatous inflammation, and necrotising glomerulonephritis. The etiology of GPA is thought to be linked to environmental and infectious triggers inciting onset of disease in genetically predisposed individuals. Antineutrophil cytoplasmic antibodies (ANCAs) play an important role in the pathogenesis of this disease, although ANCA positivity is not essential for a clinical diagnosis of GPA. The diagnosis is based on clinical manifestations of systemic vasculitis and histological evidence of necrotizing vasculitis or granulomatous inflammation.

## 2. Case Presentation

**Figure 1:**
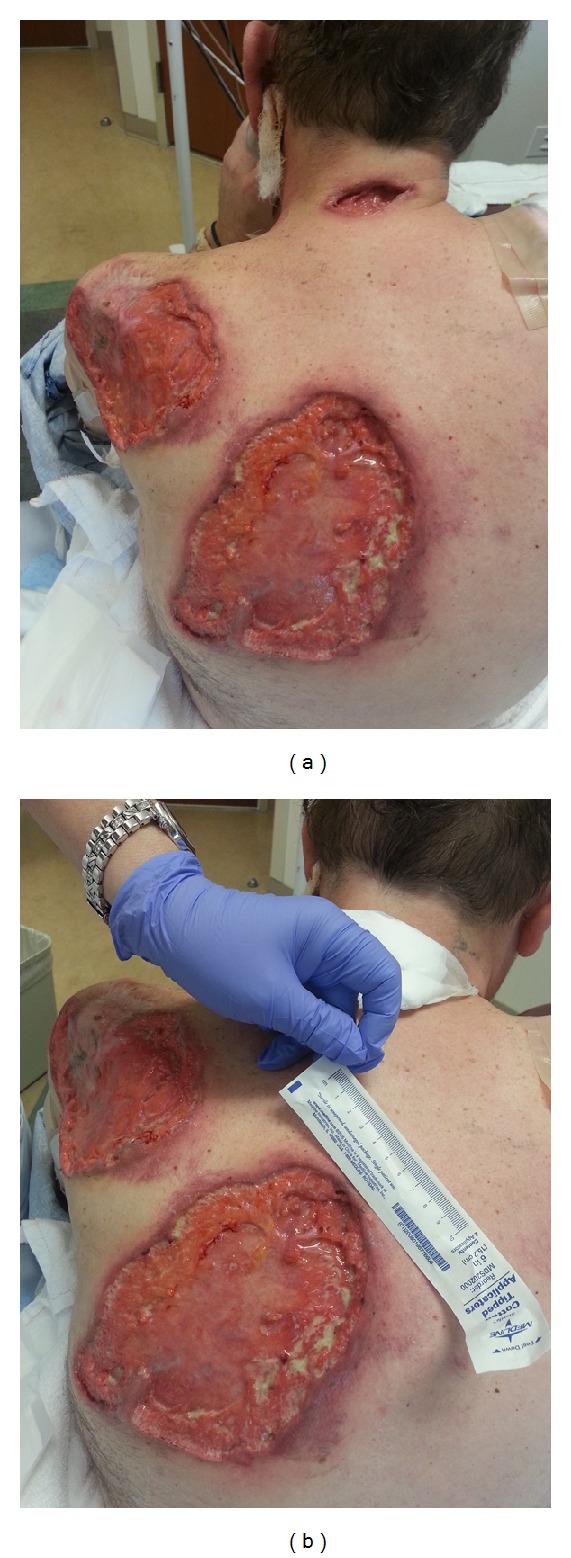
(a) and (b) Multiple large ulcers with irregular, violaceous, undermined borders and yellow necrotic centers, with the largest measuring 20 × 10 cm.

A 52-year-old Caucasian man presented with multiple large nonhealing ulcers on his upper extremities and back that started two months earlier. He stated that he was previously healthy when he bumped into a shelf at the supermarket and superficially scratched his arm. Over the next week, his wife noticed that the scratch became erythematous and increased in size. After completing treatment with a course of antibiotics, the scratched area soon became tender and black in color. A skin biopsy showed areas of acute and chronic inflammation with dermal necrosis and negative for microorganisms on stains and cultures. At the site of the biopsy purulent drainage was noted and an ulcer developed. Over the next two months, the patient developed multiple painful ulcers over the face, back, and abdomen. These ulcers all began as areas of intense erythema and tenderness with subsequent purulent drainage and ulceration (Figures 1(a) and 1(b)).

**Figure 2:**
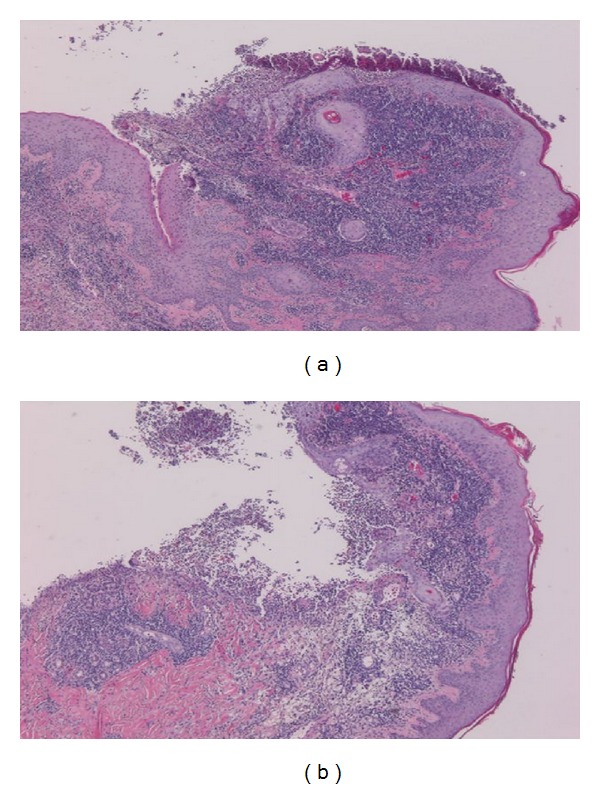
(a) and (b) (4x) Surface ulcerations with mixed acute and chronic inflammation and overlying fibrinopurulent exudate.

**Figure 3:**
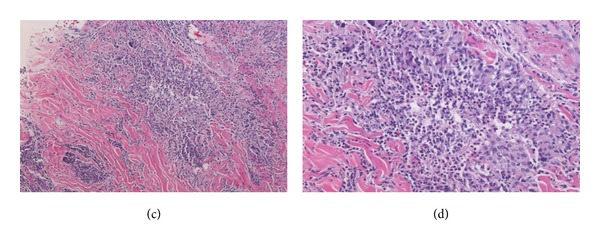
(c) (10x) and (d) (20x) Microgranulomas with suppurative necrosis. Small foci of tissue necrosis with acute inflammation, surrounded by histiocytes and multinucleated giant cells (arrows).

**Figure 4:**
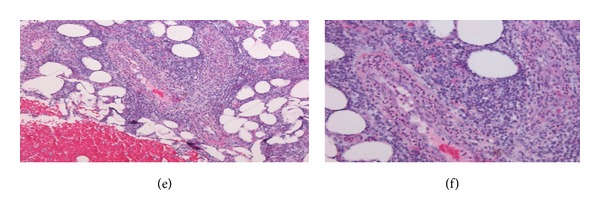
(e) (10x) and (f) (20x) Vessels in the deep dermis and subcutis are infiltrated by neutrophils and have fibrin in their walls.

Repeated microbial stains and cultures showed negative results, and repeated biopsies from several ulcers (Figures 2, 3, and 4) showed pyoderma gangrenosum-like features with foci of prominent granulomatous and neutrophilic necrotizing vasculitis with basophilic collagen degeneration. Serological testing was positive for PR3/c-ANCA. Treatment was initiated with Cyclophosphamide and steroids, but persistent nonhealing ulcers remained and became infected requiring frequent antimicrobial treatment and careful debridement.

**Figure 5:**
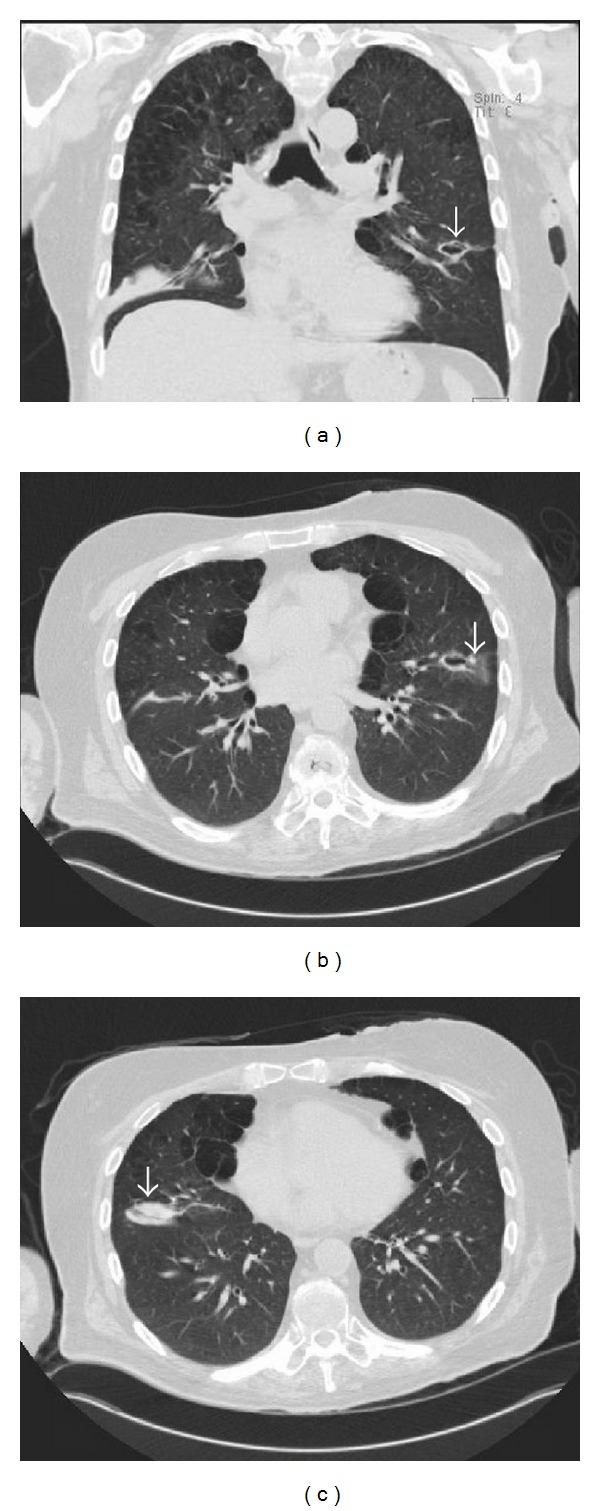
Chest CT scan. Coronal view (a) and axial views ((b) and (c)) showing a right lower lobe mass-like dense opacity with a left lower lobe cavitary lesion (arrows) and upper lobe predominant paraseptal emphysema.

Six months after the first ulcer, recurrent episodes of purulent nasal discharge and epistaxis were reported by the patient and a perforated nasal septum was seen on physical exam. Pulmonary evaluation revealed multiple lung opacities with cavitary lesions (Figure 5).

The patient underwent a bronchoscopy procedure with a bronchoalveolar lavage (BAL) from the lung segments corresponding to the cavitary and mass-like lesions, with negative results on gram stain, cultures, and acid fast bacilli staining, as well as absent growth on fungal and mycobacterial cultures. However, the BAL samples showed neutrophil predominant cells, 52% neutrophils, with no evidence of malignant cells on cytological exam, indicating an inflammatory lung disease. The clinical history and BAL results were supportive of GPA lung disease.

The patient had a modest response to treatment with Methotrexate, characterized by reduced inflammation at the ulcer margins, and repeated serological tests 3 months after initiating Methotrexate showed negative PR3/c-ANCA results; however Methotrexate was discontinued for gastrointestinal side effects and treatment with Rituximab was initiated.

A reduction in the size of ulcers and surrounding inflammation was described a few months after initiating Rituximab.

## 3. Discussion

No reliable test can identify which patients with GPA skin lesions will eventually show systemic signs and symptoms [1, 2].

The presence of compatible histologic features of GPA, cutaneous manifestations, and positive c-ANCA/PR3-ANCA serologic test results should raise clinical suspicion for the subsequent development of systemic disease. Such patients should have frequent follow-up evaluations to be screened for internal organ involvement, and appropriate management should be initiated early in the disease course. Serial tests for ANCAs are recommended because ANCA levels correlate with disease activity [3].

Differential diagnosis for refractory cases of cutaneous GPA must include indolent infectious etiologies, such as mycobacterial infections and cutaneous leishmaniasis, where biopsies also show a granulomatous inflammation [4].

The most common skin lesions are palpable purpura, necrotic ulcerations, papules and nodules with many histological patterns, leukocytoclastic vasculitis, granulomatous vasculitis, and palisading granulomas [5].

In a retrospective analysis by Daoud et al. on 244 cases of GPA, necrotizing ulcerations resembling pyoderma gangrenosum were not uncommon. Leukocytoclastic vasculitis was the most common cutaneous pathologic pattern. Findings of c-ANCA were positive in 81% of patients with cutaneous GPA [6].

Treatment strategies are tailored to the severity of the disease. They are based on published evidence of the efficacy and safety of the immunosuppressive drugs indicated to manage active vasculitis and maintain clinical remission.

Currently, hopes are raised by biological agents. Rituximab is one of the best studies of monoclonal antibodies used as an alternative treatment for patients with refractory disease [7, 8].

## 4. Conclusion

Pyoderma gangrenosum-like lesions in GPA have been rarely reported. In these cases, multiple devastating pyoderma gangrenosum-like lesions and disease activity were controlled with biologic agents, which may prove to be effective in this subcategory of patients who are refractory to standard immunosuppressive therapy.
